# Feed conversion ratio, residual feed intake and cholecystokinin type A receptor gene polymorphisms are associated with feed intake and average daily gain in a Chinese local chicken population

**DOI:** 10.1186/s40104-018-0261-1

**Published:** 2018-06-14

**Authors:** Zhenhua Yi, Xing Li, Wen Luo, Zhenqiang Xu, Congliang Ji, Yan Zhang, Qinghua Nie, Dexiang Zhang, Xiquan Zhang

**Affiliations:** 1 Department of Animal Genetics, Breeding and Reproduction, College of Animal Science, South China Agricultural University, Guangzhou, 510642 Guangdong China; 2 Guangdong Provincial Key Lab of Agro-Animal Genomics and Molecular Breeding and Key Laboratory of Chicken Genetics, Breeding and Reproduction, Ministry of Agriculture, Guangzhou, 510642 Guangdong China; 3 Wen’s Nanfang Poultry Breeding Co. Ltd, Yunfu, 527400 Guangdong China

**Keywords:** CCKAR, Chicken, FCR, RFI, SNP

## Abstract

**Background:**

The feed conversion ratio (FCR) and residual feed intake (RFI) are common indexes in measuring feed efficiency for livestock. RFI is a feed intake adjusted for requirements for maintenance and production so these two traits are related. Similarly, FCR is related to feed intake and weight gain because it is their ratio. Cholecystokinin type A receptor (*CCKAR*) plays an important role in animal digestive process. We examined the interplay of these three parameters in a local Chinese chicken population.

**Results:**

The feed intake (FI) and body weights (BW) of 1,841 individuals were monitored on a daily basis from 56 to 105 d of age. There was a strong correlation between RFI and average daily feed intake (ADFI) and a negative correlation between the FCR and daily gain (*r*_g_ = − 0.710). Furthermore, we identified 51 single nucleotide polymorphisms (SNPs) in the *CCKAR* and 4 of these resulted in amino acid mutations. The C334A mutation was specifically associated with FI and the expected feed intake (EFI) (*P* <  0.01) and significantly associated with the average daily gain (ADG) (*P* <  0.05). G1290A was significantly associated with FI and EFI (*P* < 0.05).

**Conclusion:**

FCR is apply to weight selecting, and RFI is more appropriate if the breeding focus is feed intake. And C334A and G1290A of the *CCKAR* gene can be deemed as candidate markers for feed intake and weight gain.

**Electronic supplementary material:**

The online version of this article (10.1186/s40104-018-0261-1) contains supplementary material, which is available to authorized users.

## Background

Feed expenses account for approximately 70% of chicken production costs [1]. One way to reduce fodder costs is by increasing the utilization rate of feed. The FCR and RFI are pivotal indicators for measuring poultry feed efficiency. The FCR is calculated using FI and body weight gain (BWG) and is a proportional trait that does not have a normal statistical distribution, the degree of abnormal distribution will increase with the increase of the variable coefficient of the denominator, the mean values and standard deviations have no actual statistical significance [2–4].

As a selection index, the FCR is cannot be used to determine whether FI or BWG predominate and this reduces the group selection difference and affects the efficiency of selection [5]. From a population genetic standpoint, the FCR is a moderately heritable trait and used as an indicator of the outcomes of other genetic improvements [6]. This type of selection results in the synchronous selection of FI and BWG with a population improvement bias towards high FI and high BWG. The outcome is an increase in BWG and the feed cost, and the weight of the traits in the selection index is determined by their desired gains.

In order to make up for the defects of FCR calculations, the RFI has been used as a production performance evaluation index for layer chickens since the 1970s. RFI is a measure of the feed utilization efficiency index of livestock first proposed in 1963 [7]. The RFI is the difference between the actual animal FI and its EFI determined by the growth rate and mean BW. By dividing the total energy of livestock and poultry into growth energy and maintenance energy, RFI can accurately reflect the metabolic differences among individuals in which metabolic differences are determined by genetic background [8]. The feed intake of high RFI individuals is higher than with low RFI individuals. Therefore, using the RFI as a negative selection trait is more likely to produce a population with low feed intake and high productivity.

The brain-gut axis encompasses the hypothalamus, vagus nerve, stomach and intestine and regulates eating behaviors [9–11]. Cholecystokinin (CCK) is a regulatory peptide of the brain-gut axis that is widely distributed in central and peripheral nerves and the digestive system. Its primary function is to promote gallbladder contraction and pancreatic secretion [12]. However, CCK cannot properly function without the cholecystokinin receptor (CCKR). There are two CCKR subtypes (CCKAR and CCKBR). CCKAR mainly exists in peripheral tissues and is responsible for regulating satiety and inhibiting gastric emptying [13–16]. CCKBR is distributed in the central nervous system and is involved in nerve responses [17]. Thus, the main route for the regulation of feeding by CCK is its stimulation of the vagus nerve through peripheral CCKAR. The activated vagus nerve stimulates the central nervous system to produce CCK resulting in satiety and termination of feeding behavior [18]. The expression levels of *CCKAR* in the individual brains are inversely proportional to growth rate [17]. Furthermore, *CCKAR* knockout mice have significantly higher FI levels than their wildtype counterparts [14]. Therefore, *CCKAR* influences the regulation of feed intake and growth of animals.

Chinese local chickens have several excellent production traits such as high meat quality, strong adaptation and crude feed tolerance [19]. However, their growth rates and feed utilization efficiency are low. The purpose of this study was to analyze both these parameters in Chinese local chickens, and increase growth rate and feed efficiency by selection. We calculated FCR and RFI in a Tianlu black chicken population and assessed genetic parameters and the relative selection efficiency of FCR and RFI. In addition, Xu et al. [20] performed a genome-wide association study (GWAS) and RNA sequencing on RFI of the yellow-plumage dwarf chicken line N301, and found that *CCKAR* is a potential candidate gene associated with energy improvement, so we also analyzed the associations between variation in the *CCKAR* and the phenotypes measured.

## Methods

### Animals and measurement of feeding traits

A Chinese local chicken population, a Tianlu Black chicken pure-line N416, was used for measurement of growth and feed conversion rate traits in this study. Chickens were housed in a closed type henhouse to control the temperature and illumination during the brooding period (0–35 d of age). After 35 d of age, chickens were transferred to a half-open vertical ventilation hoop henhouse, and electronic chips were placed in the middle of shank. A total of 912 male individuals were kept in three fence-separated pens (every pen had 304 birds) on one side of the henhouse, and 929 female ones were kept in three fence-separated pens (309 birds in one pen, 310 ones in other two pens) on the other side of the same henhouse. Each pen had seventeen 9ZC-5 intelligent type breeding and feeding automatic measurement stations (Guangdong Guangxing Animal Husbandry Co., Ltd., China) and 40 nipple water bowls, the 9ZC-5 stations can recognize each chicken’s electronic chip and record the FI and BW of the chicken. The chickens were fed a diet containing 12.1 MJ/kg ME and 190 g CP/kg. Daily feed intake and body weights were recorded for each bird throughout the feeding trial from 56 to 115 d of age, and this working was performed as previously described [20], as some individuals were died or the electronic chips were not identified by the 9ZC-5 stations during the feeding period, at the end of the data screening, 538 sires and 682 dams contributed to these males and females under feed recording finally. For reducing cost, we selected 527 individuals from these 1,220 individuals for sequencing, these individuals are randomly selected.

### Calculation of RFI and FCR

The RFI calculation was based on a previously described model [21]. The experimental period was conducted during the rapid growth period of the animals so the individual BW and ages were linearly related. The regression equation was

1 $$ \mathrm{BW}=\mu +a\times \mathrm{DOT}+e $$

where *μ* is the intercept, *a* is the regression coefficient, DOT was the day of testing and *e* is the residual. A DOT value of 25 is put into Eq. 1 to get the mid-test body weight (MBW) and the mid-test metabolic body weight, (MMBW) = MBW^0.75^.

The MMBW and ADG of each bird were used as independent variables to establish the linear regression models,

2 $$ \mathrm{ADFI}=\mathrm{FI}/\mathrm{DOT} $$

3 $$ \mathrm{ADFI}={b}_0+{b}_1\times \mathrm{MMBW}+{b}_2\times \mathrm{ADG}+e $$

and

4 $$ \mathrm{RFI}=\mathrm{ADFI}-\left({b}_0+{b}_1\times \mathrm{MMBW}+{b}_2\times \mathrm{ADG}\right) $$

In these models, ADFI is the average of the daily feed intake of individuals during the period of the experiment, *b*_0_ is the intercept, *b*_1_ and *b*_2_ are partial regression coefficients for MMBW and ADG, respectively, and *e* was the residual and the RFI is the *e* of these models. The experimental data were then divided into two groups by sex and two equations were developed using ADFI, MBW and an ADG estimate of the EFI.

FCR is the net feed consumption of livestock unit weight gain. The FCR for each individual was estimated based on the ratio between unit weight gain and feed consumption.

### Genetic parameters estimation

We constructed the multi-trait animal model:

5 $$ Y= Xb+ Za+e $$

to obtain estimates of the phenotypic and genetic (co)variance and heritability, and this model was based on the restricted maximum likehood method of the DMU statistical package [4, 20, 22]. *Y* is the vector under observation, *X* and *Z* was incidence matrices, *b* is a vector of fixed effects (including two gender levels and 6 pen levels), *a* is the vector of the animal additive genetic effect and *e* is the vector of random residuals.

### Relative selection efficiency estimation

Relative selection efficiencies were used to compare the expected effects of selection on FCR and RFI on other growth traits. In order to estimate the correlated response to selection on feed efficiency, we referred to previous report [23] and constructed the following equation:

6 $$ C\Delta G/\Delta G=\left({h}_1/{h}_2\right)\times {r}_{A1A2} $$

where *C∆G* represents the FCR and RFI for indirect selection efficiencies on growth. *∆G* represents direct selection efficiencies that are induced through selection of growth and other primary traits. *r*_*A*1*A*2_ represents genetic correlation coefficients between primary and secondary traits and *h*_1_ and *h*_2_ represents the heritability of primary and secondary traits, respectively.

### DNA manipulations and genotyping procedure

DNA was extracted from whole blood using the EZNA Blood DNA Kit (OMEGA Biotek, Doraville, GA). PCR primers for chicken *CCKAR* (NCBI Gene ID: 422801) were designed using Primer Premier 5.0 software (http://www.premierbiosoft.com). Primers were synthesized by Shanghai Jierui Biological Technology. Five pairs of primers were designed to perform mixed pool sequencing for an initial screen of SNPs in *CCKAR* (Additional file 1: Table S1). For this purpose, data from the 10% highest- and 10% lowest-ranked RFI birds (each group had 48 individuals) were pooled randomly in 12 pools for sequencing. PCR was performed in a 40-μL volume consisting of 10 pmol of each primer, 20 μL of 2× Easy Taq SuperMix (Beijing TransGen Biotech Co., Ltd., China) and 50 ng of genomic DNA. The PCR procedure was as follows: 94 °C for 3 min, followed by 36 cycles of 94 °C for 30 s, 55–60 °C for 30 s and 72 °C for 2 min and a final step of 72 °C for 10 min. The PCR products were sequenced by Beijing Tsingke Biological Technology, China.

SNP genotyping was performed using the SNaPshot method [24]. Based on the preliminary SNP screening, 5 pairs of primers were designed for the 10 SNPs. P1 was designed for G176A, G219A, C334A, C448T, P2 for G1290A, P3 for C5818T, G6058A, A6163G, P4 for T3325C, and P5 for G6768A. PCR was performed in 15 μL, consisting of 3 pmol of each primer, 0.3 μL of 2× Easy Taq SuperMix, 30 ng of genomic DNA, 1.5 μL MgCl_2_, 0.3 μL dNTP and 1.5 μL 10× buffer. The PCR procedure was as follows: 94 °C for 3 min, followed by 36 cycles of 94 °C for 15 s, 55 °C for 30 s and 72 °C for 30 s with a final step of 72 °C for 10 min. The PCR products were purified by using ExoI (Thermo Fisher Scientific Inc., USA) and FastA (Thermo Fisher Scientific Inc., USA). Genotyping was performed using a commercial kit (SNaPshot Multiplex Kit, Thermo Fisher Scientific Inc., USA) using 2 μL of purified products as directed by the specification, the purified primers were showed in Additional file 1: Table S1.

### Linkage disequilibrium and haplotype analysis

Linkage disequilibrium (LD) and haplotype analysis for this study population was performed using SHEsis software (http://analysis.bio-x.cn/myAnalysis.php). We excluded polymorphic sites that did not conform to the Hardy-Weinberg Equilibrium (HWE) for the analysis. D´ was a standardized Lewontin LD coefficient as the evaluation criterion for LD. │D´│ > 0.75 represented a strong LD between each pair of polymorphic sites. A full-precise-iteration algorithm was used for haplotype analysis. This was based on the equation:

7 $$ N\kern0.28em (11)=2\kern0.28em N\left(11/11\right)+N\left(12/11\right)+N\left(11/12\right)+P\left[\left(11/22\right)\kern0.28em |\kern0.28em \left(\mathrm{XX}\right)\right]\ast N\left(\mathrm{XX}\right) $$

where *N*(11) represents the number of “11″ haplotype, *N*(12/11) represents the number of samples that carried “12″ haplotype on one chromosome and “11″ on another, *N*(XX) represents the number of samples carrying both “1/2″ genotypes at the two loci that was ambiguous for haplotype recognition.

### Association analysis between SNPs and characters

The PROC-GLMR algorithm [25] was used to analyze the correlation of SNPs, FI and related traits. A statistical model was developed as follows:

8 $$ Y=\mu +G+F+S+e $$

where *Y* is the observed value of growth traits, *μ* is the population mean value of growth traits, *G* is the effect of genotype on growth performance, *F* is the fixed effect of family, *S* is the gender effect (two levels), and *e* is the random error effect corresponding to the observed value. The evaluation of traits among individuals with different genotypes was performed using the paired comparison method of ANOVA using SPSS version 19.0 software.

## Results

### BW, ADFI, MBW and RFI of Chinese local chickens

**Fig. 1:**
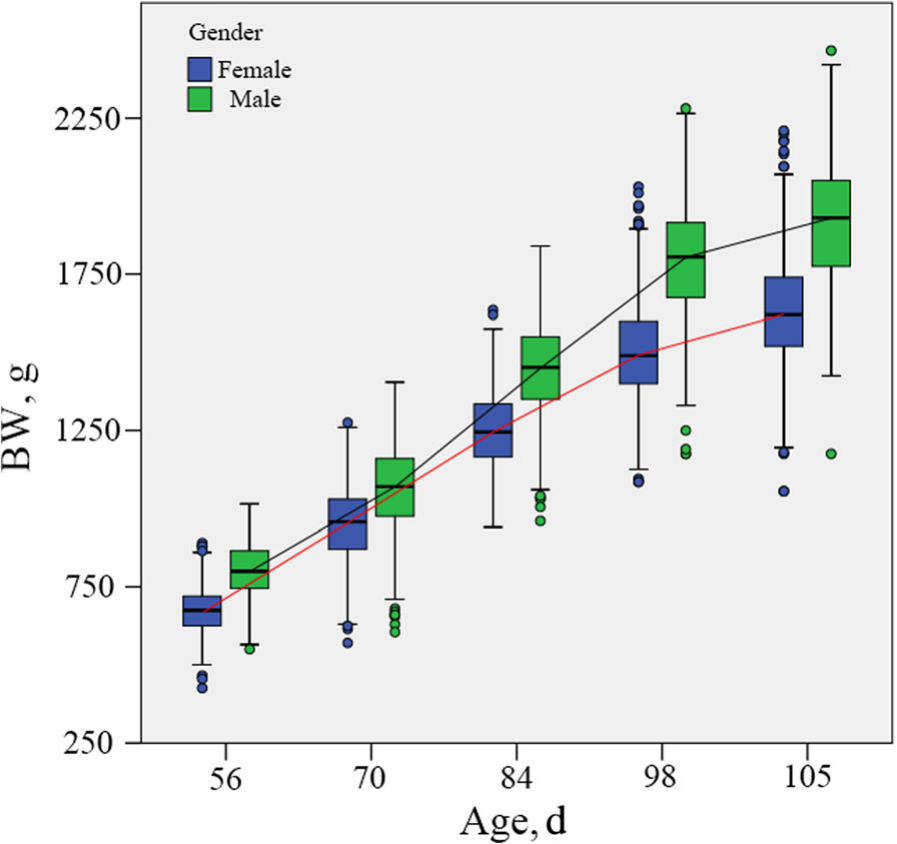
The weekly average weight of chickens during the experimental period

At the beginning of the feeding trial, the average BWs of male and female individuals at 56 d of age were 802 ± 86 g and 671 ± 73 g respectively. The average BWs at 115 d of age were 1,915 ± 191 g for male and 1,632 ± 175 g for females (Fig. 1). During the study period, the ADFI of males and females were 22.7 ± 3.31 g and 19.6 ± 2.97 g respectively. According to the Eq. 1, we obtained the MBW. The average MBW of male and female chickens was 1,426 ± 142 g and 1,182 ± 123 g, respectively. The determination coefficients of male and female individuals were 0.409 and 0.545, respectively. Equations were as follows,

9) $$ \mathrm{EFI}=0.381\kern0.28em {\mathrm{MBW}}^{0.75}+1.09\kern0.28em \mathrm{ADG}-5.14\kern0.45em \Big(\mathrm{for}\ \mathrm{male}, $$

10) $$ \mathrm{EFI}=0.069\kern0.28em {\mathrm{MBW}}^{0.75}+1.48\kern0.28em \mathrm{ADG}-37.8\kern0.41em \Big(\mathrm{for}\ \mathrm{female}, $$

### Performance of growing and feed efficiency Chinese local chickens with different RFI values

**Fig. 2:**
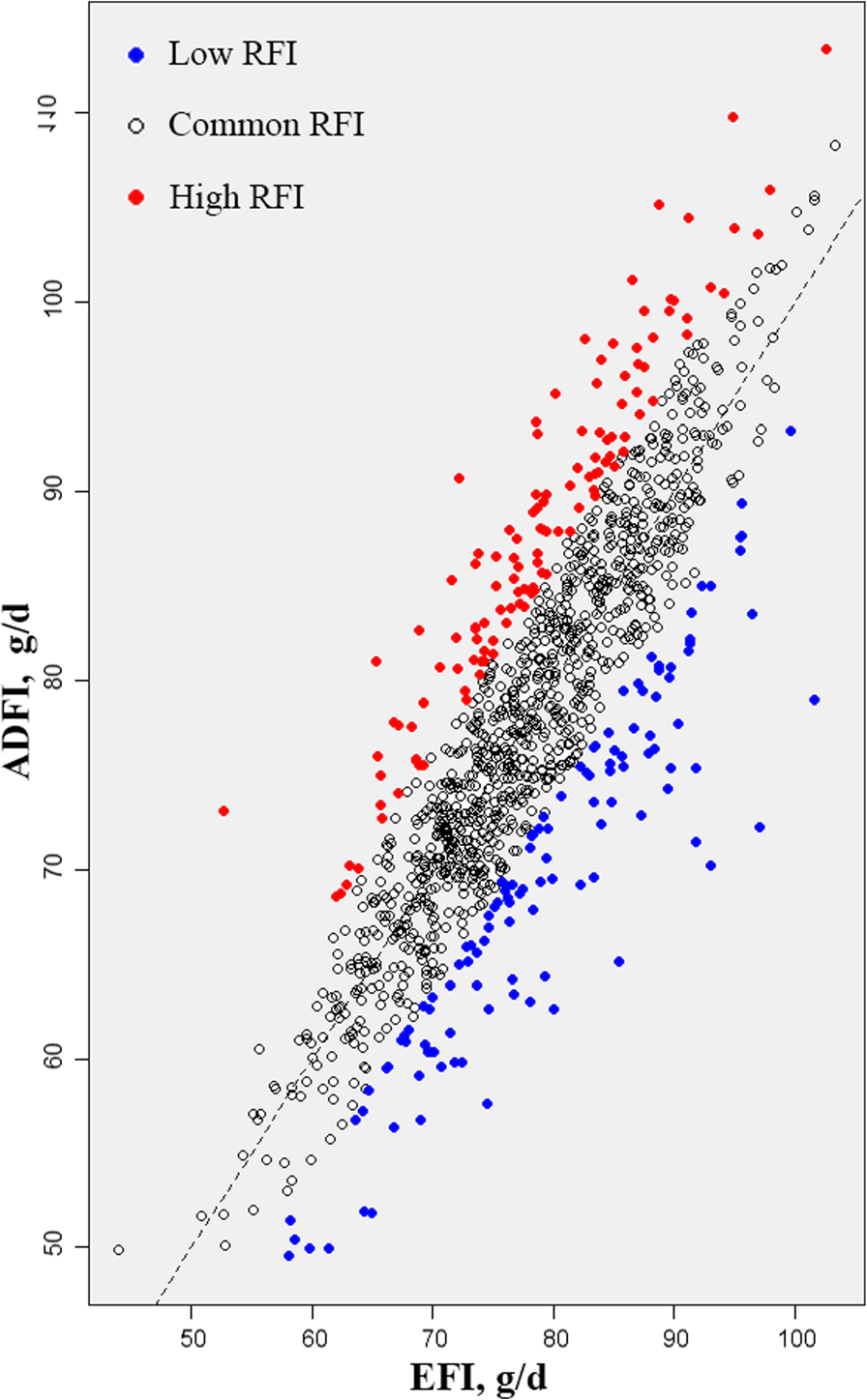
Comparison between ADFI and EFI. Points above the dotted line represent low RFI individuals with high energy utilization and below the line represent high RFI individuals with poor energy utilization

We observed growth performance and feed efficiency in chickens that possessed different RFI values and defined two groups of individuals with the 10% lowest and the 10% highest RFI values. We found that ADG, MBW, EFI and BW15 differences between the two groups were not significant. However, FCR and ADFI were significantly different in the higher and lower RFI group with a difference of 0.90 for FCR and a difference of 17.90 g for ADFI. The BW8 of the lower RFI group was significant (*P* < 0.05) less than the higher RFI group (Table 1). In addition, the ADFI in the higher RFI group was more than the predicted value whereas the situation with the lower RFI group was reversed (Fig. 2). This indicated that the energy utilization rate of the lower RFI group exceeded the higher RFI group.

**Table 1:** Traits of 10% lowest RFI and 10% highest RFI individuals

Traits	Highest	Lowest	*P*-value
RFI, g	9.13 ± 2.87	− 9.66 ± 3.66	–
FCR	4.20 ± 0.608	3.30 ± 0.360	< 0.01
ADG, g	21.2 ± 3.60	21.4 ± 4.18	0.666
ADFI, g	87.2 ± 9.88	69.3 ± 9.15	< 0.01
EFI,g	78.1 ± 9.28	79 ± 9.75	0.464
MMBW, g	1197 ± 131	1212 ± 122	0.377
BW8, g	742 ± 110	709 ± 97.7	0.013
BW15, g	1779 ± 240	1756 ± 252	0.472

### Heritability of the FCR, RFI and related traits

We estimated heritability of 7 characteristics. RFI heritability was 0.282 in the range 0.21–0.49 and similar results have been previously reported [26]. The FCR heritability was 0.312 that was between 0.2 and 0.8 values previously reported [27] (Table 2).

**Table 2:** Heritability characteristics

Character	Heritability (estimate ± standard deviation)
FCR	0.312 ± 0.067
RFI, g	0.282 ± 0.066
BW at 56 d of age, g	0.305 ± 0.075
BW at 115 d of age, g	0.395 ± 0.077
ADG, g	0.383 ± 0.074
MMBW, g ^0.75^	0.321 ± 0.005
ADFI, g	0.288 ± 0.068

### Correlation of FCR and RFI with other traits

We calculated the genetic and phenotypic correlation coefficients between RFI, FCR and other traits. Firstly, there was a strong correlation between RFI and FCR in both heritability and phenotype. A 0.693 genetic correlation coefficient between the two was in the 0.6–0.7 range as has been previously reported [8, 28]. Although the phenotypic correlation coefficients between RFI and ADG or MMBW were 0, the genetic correlation coefficient between RFI and the two traits were − 0.198 and − 0.125 (Table 3). This demonstrated that the RFI was independent of these two phenotypic traits. and this result was consistent with previously reported results [8, 26, 29].

**Table 3:** Genetic correlations and phenotypic correlations between FCR, RFI and other traits

Character	RFI, g	FCR
FCR	0.693 ± 0.094^a^	–
	0.653** ^b^	–
BW at 56 d of age, g	0.0281 ± 0.183	0.261 ± 0.166
	0.111**	0.044
BW at 115 d of age, g	−0.163 ± 0.167	− 0.496 ± 0.132
	0.050**	− 0.395**
ADG, g	−0.198 ± 0.165	−0.710 ± 0.092
	0	−0.561**
ADFI, g	0.334 ± 0.154	−0.143 ± 0.173
	0.501**	−0.11
MMBW, g	−0.125 ± 0.178	−0.176 ± 0.173
	0	−0.163**

^a^Genetic correlations, *r*_g_ = Mean ± standard deviation; ^b^ Phenotypic correlations, *r*_p_; ** *P* < 0.01

Compared to the RFI, the genetic and phenotypic associations between FCR and ADG were more close and all showed a strong negative correlation (*r*_g_ = − 0.710, *r*_p_ = − 0.561). This meant that the FCR had a greater impact on ADG and selected for low FCR individuals. This would results in an ADG increase. The genetic and phenotypic correlation coefficients between RFI and ADFI were 0.334 and 0.501, and that two correlation coefficients between FCR and ADFI were *r*_g_ = − 0.143 and *r*_p_ = − 0.11, so the correlation coefficients of between FCR and ADFI were both much less than that between RFI and ADFI (Table 3). This suggested that choosing RFI was more beneficial for individual consumption than FCR. Overall, the FCR was primarily related to growth traits such as ADG, BW while RFI was related to energy metabolism traits such as FI.

### Selection efficiency of RFI and FCR with other related traits

We obtained selection efficiencies of RFI and FCR with five related traits by calculation (Table 4). RFI had a strong positive selection effect to ADFI (0.327), but it had little effect to body weight at 56 d of age (BW8) (0.026). In addition, RFI had negative selection effect to body weight at 105 d of age (BW15), ADG and MMBW, and the effect coefficient were − 0.116, − 0.108 and − 0.174, respectively. For FCR, it only had positive selection effect to BW8 (0.268), and the remaining four traits were negative to selective effect, especially the negative selection effect to MMBW, ADG, BW15, the selection efficiency were as high as − 0.691, − 0.680 and − 0.392, respectively, and it had a slight negative effect to ADFI (− 0.155). Therefore, genetic selection of individuals with lower RFI could reduce ADFI and increase ADG. Furthermore, the selection for lower FCR would lead to better weight gain at a cost of slightly higher feed intake, but the genetic correlation considering standard error is not significantly different from 0, so not necessarily any change in feed intake, so the selection direction of RFI and FCR was consistent.

**Table 4:** Selective reactions between RFI, FCR and related traits

Character	RFI, g	FCR
BW at 56 d of age, g	0.026	0.268
BW at 115 d of age, g	−0.116	− 0.392
ADG, g	−0.108	− 0.680
MMBW^0.75^, g	−0.174	− 0.691
ADFI, g	0.327	−0.155

### Association of *CCKAR* with chicken growth and feed conversion rate

**Fig. 3:**
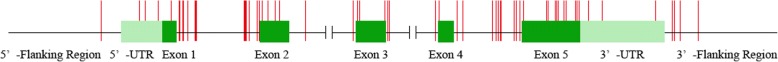
Distribution of 51 SNPs in the chicken *CCKAR* gene The pale green regions represent untranslated regions; the dark green regions represent exons. The red regions represent the SNPs sites. The first nucleotide of the translation start codon was designated + 1

We screened 51 mutation sites in the *CCKAR* gene. Five mutation sites were in flanking regions, five were in untranslated regions, 31 in introns and 10 in coding regions. Four SNPs generated amino acid substitutions (Fig. 3, Additional file 2: Table S2).

**Fig. 4:**
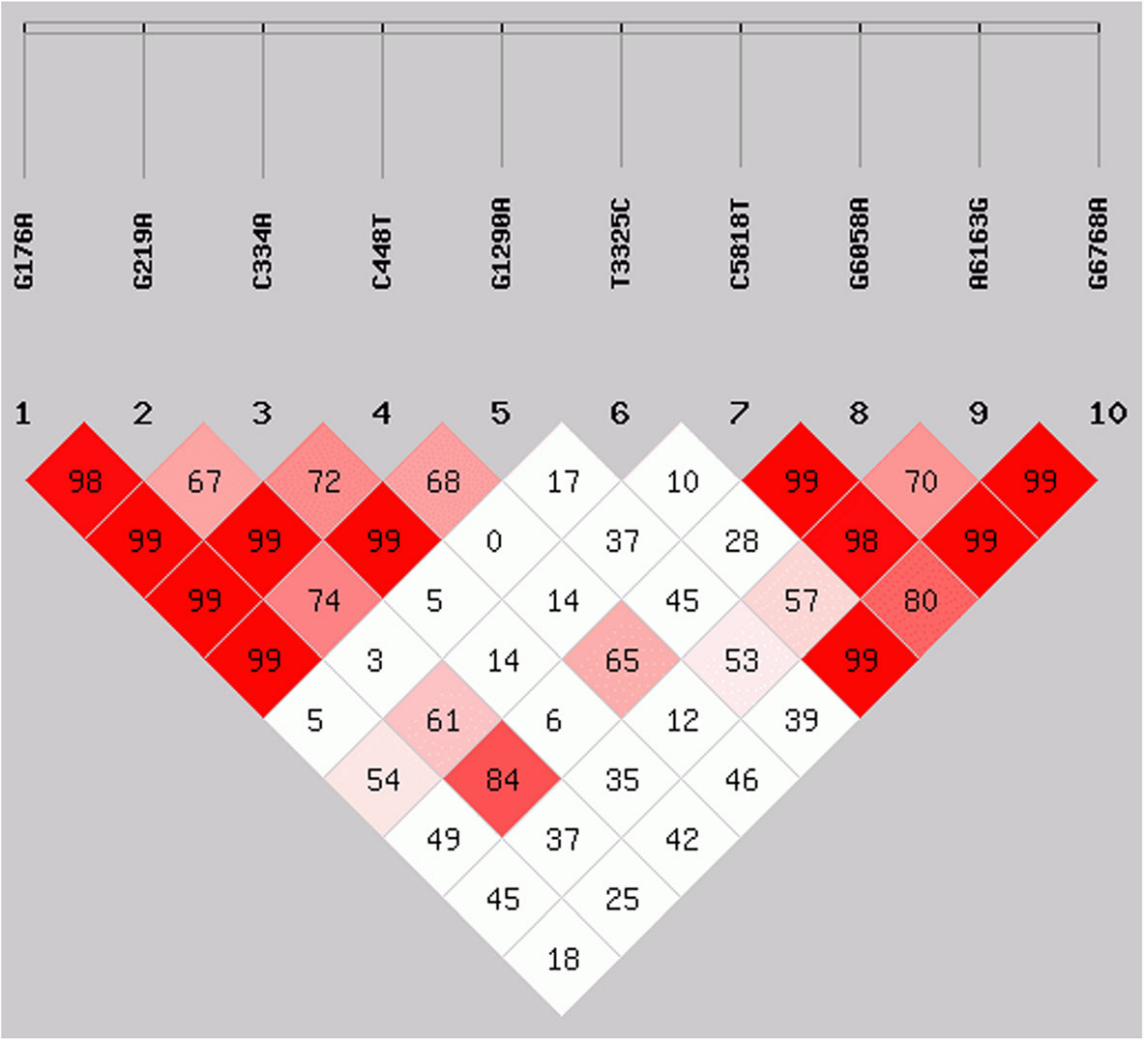
Linkage disequilibrium analysis of SNPs in the study population

In order to see if these SNPs lead to amino acid mutations, we selected the SNPs of coding regions for HWE, LD and haplotype analyses. We found that the allele frequencies of these 10 SNPs followed the Hardy-Weinberg equilibrium rule (*P* > 0.05) (Additional file 3: Table S3). There was a strong LD between SNPG176A-G219A and G176A-G1290A (D′ > 0.75 and *r*^2^ > 0.6) as well as between SNP G219A-C448T and C334A-G1290A (D′ > 0.75 and *r*^2^ > 0.3) (Fig. 4, Table 5). The LD between each SNP pair was incomplete so we used all 10 SNPs to correlate chicken growth and feed conversion rate for the next experiments.

**Table 5:** Haplotype analysis *r*^2^ values

*r* ^2^	G219A	C334A	C448T	G1290A	T3325C	C5818T	G6058A	A6163G	G6768A
G176A	0.704	0.297	0.247	0.694	0.001	0.085	0.127	0.128	0.003
G219A	─	0.187	0.343	0.531	0.001	0.150	0.265	0.063	0.008
C334A	─	─	0.433	0.428	0.002	0.004	0.001	0.024	0.050
C448T	─	─	─	0.168	0.000	0.004	0.055	0.016	0.072
G1290A	─	─	─	─	0.022	0.059	0.076	0.125	0.018
T3325C	─	─	─	─	─	0.004	0.020	0.098	0.175
C5818T	─	─	─	─	─	─	0.150	0.175	0.039
G6058A	─	─	─	─	─	─	─	0.412	0.043
A6163G	─	─	─	─	─	─	─	─	0.052

We utilized GLM function to analyze the associations of the 10 SNPs with RFI, FCR, FI, ADFI, and ADG. The association of these SNPs with RFI and FCR were not significant. However, C334A was highly and significantly associated with FI and EFI (*P* < 0.01) and significantly associated with ADG (*P* < 0.05). The FI and EFI of individuals with the CC genotype were significantly lower than with the other two genotypes (*P* < 0.05). However, the ADG in this CC genotype group was significantly lower than in the AA group (*P* < 0.05). G1290A was significantly associated with FI and EFI (*P* < 0.05) in which the FI and EFI associated with the GG genotype were significantly lower than those associated with the AA genotype (*P* < 0.05) (Table 6).

**Table 6:** Association between SNPs and other traits

SNP	Traits	Genotype		
C334A		AA (*n* = 59)	AC (*n* = 220)	CC (*n* = 248)
	FI	3.921 ± 0.480^a^	3.922 ± 0.523^a^	3.753 ± 0.493^b^
	EFI	80.232 ± 8.589^a^	79.543 ± 8.762^a^	76.965 ± 8.40^b^
	ADG	21.546 ± 3.195^a^	21.506 ± 3.240^ab^	20.635 ± 3.565^b^
G1290A		AA (*n* = 145)	AG (*n* = 263)	GG (*n* = 119)
	FI	3.910 ± 0.519^a^	3.835 ± 0.501^ab^	3.776 ± 0.515^b^
	EFI	79.730 ± 8.424^a^	78.256 ± 8.943^ab^	77.130 ± 9.300^b^

^a,b^Different letters represent significant differences (*P* < 0.05), otherwise there were no differences found. The association was estimated using the LSD method. *α* = 0.05

## Discussion

The classical breeding index FCR has been studied extensively in chickens [30]. However, FCR is a nonlinear and non-normally distributed complex trait formed by the FI / BWG ratio. As FCR not only without real average value or variance, but also the non-normality will be raised with the increase of variance of BWG, thus, it is difficult to reflect the true efficiency of feed utilization [31]. As the intensity of selection increases, the selection pressure will move towards FI. When the phenotypic correlation coefficient between FCR and FI or BWG is increased, the genetic advantage of FCR as the selection trait will be reduced, and the selection effect is not as good as the selection of FI or BWG. Famula and Van Vleck [32] have reported that continuous selection for low FCR individuals can improve feed utilization efficiency and increase BWG, but will leads to genetic progress more slowly and will increase the feed consumption [33]. If individuals with low FCR in the same BWG were selected, it will be more beneficial to the selective process.

As a new indicator of the efficiency of feed utilization, the RFI has been widely used in chicken [34], sheep [35] and beef cattle breeding [36]. RFI is a phenotypic measurement independent of metabolic weight and BWG [31]. In this study, the phenotypic correlation between RFI and MBW, ADG was zero, genetically independent of metabolic weight and BWG and consistent with other studies [37]. Therefore, RFI can preferably reflect the energy level needed for broiler growth maintenance [38]. Individuals with low maintenance and high growth can be bred with RFI as a selective trait.

As the FCR, ADFI and BW8 of higher RFI group were extremely significant higher (*P* < 0.01) than the lower RFI group. This demonstrated that the FI in the lower RFI individuals were less than the higher RFI group and that their growth performance was also better. These low RFI individuals can achieve a greater feed efficiency.The reason may be that up-regulated genes were associated with energy metabolism, cell proliferation and fat metabolism [39]. Previous studies have demonstrated that individuals with a low RFI can maintain growth by increasing protein storage and glutamate synthesis, and achieve higher feed utilization and reduce nitrogen content in excreta [20, 40]. Individuals with high RFI were more likely to respond to stress than individuals with low RFI [41, 42]. Therefore, breeding low RFI individuals is more beneficial to an improvement in feed efficiency for this chicken population.

FCR and RFI belong to the mid- and high- heritability of traits. The heritability of 35–42 d of age Arkansas chickens was reflected by FCR and RFI values of 0.41 and 0.42 respectively [29]. The heritability of FCR and RFI in the slow type commercial broiler chickens were 0.33 and 0.45 [43], and the heritability of FCR and RFI in the yellow-plumage dwarf chickens were 0.216 and 0.354 [20], respectively. Different varieties of chickens showed different heritability of the FCR and RFI and the RFI heritability was always greater than the FCR.

In this study, the relative selection efficiency between FCR and ADG, MMBW and ADFI were − 0.680, − 0.691 and − 0.155, respectively. From these data, choosing the low FCR individuals will have a significant positive effect on ADG, MMBW, and ADFI. However, the relative selection rates of RFI for ADG, MMBW and ADFI were − 0.108, − 0.174 and 0.327, respectively. This will have a positive effect on reducing ADFI if low RFI individuals are selected and will slightly increase the ADG. Therefore, FCR is more suitable as a reference index to select weight related traits. If the breeding goal is to reduce FI, it is more appropriate to select RFI as the selection index.

Multiple genes control the chicken FI trait [44]. The *CCKAR* gene is located on chromosome 4 and plays an important regulatory role on feeding behaviors [14, 45]. *CCKAR* is only expressed in low RFI individuals, and CCK can be combined with CCKAR to make the individual feel full. While *CCKAR* is not expressed in high RFI individuals, this results CCK lacks the receptor and can’t exert the function of suppressing appetite, this would lead to an FI increase [20, 23]. For these reasons, we studied the relationship between *CCKAR* gene polymorphisms and feed efficiency in these experiments.

The principle novelty of this research lies in the association between variation in *CCKAR* gene and the phenotypes measured. Although the 10 *CCKAR* SNPs were not significantly associated with either RFI or FCR, C334A and G1290A were associated with FI, EFI and ADG. The AA type was significantly higher than the CC type for FI as well as ADG at the C334A locus (*P* < 0.05). The AA type was significantly higher than the GG type at FI at locus G1290A (*P* < 0.05). Furthermore, the FCR has a strong negative selectivity on ADG, and a slight negative selectivity towards ADFI. RFI has a strong positive selectivity for ADFI but a slight negative selectivity on ADG.

## Conclusions

The heritability of FCR and RFI from chickens from 56 to 105 d of age were moderate. RFI was phenotypically independent of daily gain and medium metabolic weight but there were certain genetic correlations. The FCR had a higher relative selective efficiency than RFI on weights. 51 SNPs were found in the *CCKAR* gene and four resulted in amino acid changes. However, only C334A and G1290A were significantly associated with the daily feed intake and daily gain.

## Additional files

Additional file 1: **Table S1.** Primer sequences used in this study. (DOCX 18 kb)

Additional file 2: **Table S2.** SNPs in the *CCKAR* gene of Tianlu Black Chickens. (DOCX 20 kb)

Additional file 3: **Table S3.** SNP genotypes, allelic frequencies and HWE. (DOCX 18 kb)

## Abbreviations

ADFI: Average of the daily feed intake
ADG: Average daily gain
AI-REML: Average information REML
BW: Body weight
BW15: Body weight at 15 week of age
BW8: Body weight at 8 week of age
BWG: Body weight gain
CCK: Cholecystokinin
CCKAR: Cholecystokinin A receptor
CCKBR: Cholecystokinin B receptor
CCKR: Cholecystokinin receptor
CP: Crude protein
d of age: Day of age
DOT: Day of testing
EFI: Expected feed intake
FCR: Feed conversion ratio
FI: Feed intake
HWE: Hardy-Weinberg Equilibrium
LD: Linkage disequilibrium
MBW: Mid-test body weight
ME: Metabolic energy
MMBW: Mid-test metabolic body weight
RFI: Residual feed intake
SNP: Single nucleotide polymorphism
