# Supplementary material for: Feed conversion ratio, residual feed intake and cholecystokinin type A receptor gene polymorphisms are associated with feed intake and average daily gain in a Chinese local chicken population
Source: J Anim Sci Biotechnol. 2018 Jun 14;9:50. doi: 10.1186/s40104-018-0261-1 (PMC6000933; doi:10.1186/s40104-018-0261-1)
Supplement: Supplementary file 1 — Table S1. Primer sequences used in this study. (DOCX 18 kb) [file 40104_2018_261_MOESM1_ESM.docx]

**Table S1.** Primer sequences used in this study

| **Primer names** | **Primer sequence (5′ → 3′)** | **Amplification length， bp** | **Application** |
| --- | --- | --- | --- |
| **CCKAR1 F** | GCACTTCGCTAAATGAGA | 1007 | For pool sequencing, which aimed to initially screen out SNPs of the *CCKAR* . |
| **CCKAR1 R** | TGTACCCAATGGTCCTAC |  |  |
| **CCKAR2 F** | AGTTGTATCTGGCTCTTCA | 815 |  |
| **CCKAR2 R** | GGTCGCTGCTAATAGTTC |  |  |
| **CCKAR3 F** | GGCACTAGAGCAATGAGC | 664 |  |
| **CCKAR3 R** | AAAGAGTCGAAATGTAAGC |  |  |
| **CCKAR4 F** | GGGCAGGAGAAATGTAGG | 690 |  |
| **CCKAR4 R** | AACGGAATCACCTCAGTCAA |  |  |
| **CCKAR5 F** | TGGTGCAAAGCTGGGACA | 1812 |  |
| **CCKAR5 R** | GCTCGGTAGAACAGCAGTAATC |  |  |
| **P1 F** | GCCAGTAACAGAAGAGGGTG | 572 | For SNPs genotyping. |
| **P1 R** | CTGAACTCTTTCCTGCCTGT |  |  |
| **P2 F** | TCCGGATCCTGCTGTATTGT | 173 |  |
| **P2 R** | AATGAGGGTGAATGGCATGC |  |  |
| **P3 F** | AGCTCTTCTGCCAACCTGAT | 607 |  |
| **P3 R** | GAGCCCTCTTCCTCCTTTGT |  |  |
| **P4 F** | ATCAGCACGGAGACCAAGAC | 338 |  |
| **P4 R** | CACCAGGTAGCAGCAATCAC |  |  |
| **P5 F** | GCATTTTGGGGTGGGGAGAA | 157 |  |
| **P5 R** | CATAGGTTATTGCCAGGTGA |  |  |
| **G176A** | CTGCTTTTCTAAACTGCCTCGT |  | For extent genotyping of the purified PCR products |
| **G219A** | GTGCAAGTAAGCTCTTTAACCA |  |  |
| **C334A** | TTTTTTTTTTTAGTTACAAACCAGCCTTCTTC |  |  |
| **C448T** | TTTTTTTTTTTTTTTTTTTTAAGGTGGATCATCCACACAGTA |  |  |
| **G1290A** | ATCAGCACAGTGATGACCAGAA |  |  |
| **T3325C** | CAACAAGGTTGAATGTAGACAC |  |  |
| **C5818T** | ATGCGCGCCAGGCGTTCACGCT |  |  |
| **G6058A** | TTTTTTTTTTAAGAGGAGGGGAAGACGACAAG |  |  |
| **A6163G** | TTTTTTTTTTTTTTTTTTTTCACAGAAATTCAAGTGGACATC |  |  |
| **G6768A** | TTTTTTTTTTACTGATGACTTCAAATGTGGAC |  |  |
