# Supplementary material for: Feed conversion ratio, residual feed intake and cholecystokinin type A receptor gene polymorphisms are associated with feed intake and average daily gain in a Chinese local chicken population
Source: J Anim Sci Biotechnol. 2018 Jun 14;9:50. doi: 10.1186/s40104-018-0261-1 (PMC6000933; doi:10.1186/s40104-018-0261-1)
Supplement: Supplementary file 2 — Table S2. SNPs in the CCKAR gene of Tianlu Black Chickens. (DOCX 20 kb) [file 40104_2018_261_MOESM2_ESM.docx]

**Table S2.** SNPs in the CCKAR gene of Tianlu Black Chickens

| **SNP** | **Region** | **rs number** | **Amino acid mutation** |
| --- | --- | --- | --- |
| A-818G | 5' Flanking Region | - | - |
| G176A | 5' UTR | [rs80663645](https://www.ncbi.nlm.nih.gov/projects/SNP/snp_ref.cgi?rs=80663645) | - |
| G219A | 5' UTR | [rs313597193](https://www.ncbi.nlm.nih.gov/projects/SNP/snp_ref.cgi?rs=313597193) | - |
| C334A | 5' UTR | [rs313822901](https://www.ncbi.nlm.nih.gov/projects/SNP/snp_ref.cgi?rs=313822901) | - |
| C448T | Exon 1 | [rs314084118](https://www.ncbi.nlm.nih.gov/projects/SNP/snp_ref.cgi?rs=314084118) | F - L |
| T515C | Intron 1 | [rs734635245](https://www.ncbi.nlm.nih.gov/projects/SNP/snp_ref.cgi?rs=734635245) | - |
| T516G | Intron 1 | [rs738006715](https://www.ncbi.nlm.nih.gov/projects/SNP/snp_ref.cgi?rs=738006715) | - |
| T538C | Intron 1 | [rs731807150](https://www.ncbi.nlm.nih.gov/projects/SNP/snp_ref.cgi?rs=731807150) | - |
| A539T | Intron 1 | [rs735177662](https://www.ncbi.nlm.nih.gov/projects/SNP/snp_ref.cgi?rs=735177662) | - |
| C551A | Intron 1 | [rs314558566](https://www.ncbi.nlm.nih.gov/projects/SNP/snp_ref.cgi?rs=314558566) | - |
| A580C | Intron 1 | [rs314947602](https://www.ncbi.nlm.nih.gov/projects/SNP/snp_ref.cgi?rs=314947602) | - |
| C650T | Intron 1 | - | - |
| T656A | Intron 1 | - | - |
| A657T | Intron 1 | - | - |
| T1086G | Intron 1 | [rs733514605](https://www.ncbi.nlm.nih.gov/projects/SNP/snp_ref.cgi?rs=733514605) | - |
| A1093C | Intron 1 | - | - |
| A1099C | Intron 1 | - | - |
| A1101T | Intron 1 | - | - |
| A1117C | Intron 1 | - | - |
| C1205T | Intron 1 | - | - |
| T1219C | Intron 1 | - | - |
| G1290A | Exon 2 | [rs314291632](https://www.ncbi.nlm.nih.gov/projects/SNP/snp_ref.cgi?rs=314291632) | V - I |
| C1619G | Intron 2 | [rs314874785](https://www.ncbi.nlm.nih.gov/projects/SNP/snp_ref.cgi?rs=314874785) | - |
| C1624T | Intron 2 | [rs314667473](https://www.ncbi.nlm.nih.gov/projects/SNP/snp_ref.cgi?rs=314667473) | - |
| A3279G | Intron 2 | [rs314888442](https://www.ncbi.nlm.nih.gov/projects/SNP/snp_ref.cgi?rs=314888442) | - |
| T3325C | Exon 3 | rs31527861 | Same sense mutation |
| T3343C | Exon 3 | [rs315131298](https://www.ncbi.nlm.nih.gov/projects/SNP/snp_ref.cgi?rs=315131298) | Same sense mutation |
| G3587A | Exon 3 | [rs31574411](https://www.ncbi.nlm.nih.gov/projects/SNP/snp_ref.cgi?rs=315744114) | - |
| A3601G | Intron 3 | rs315844714 | - |
| C4832T | Intron 3 | [rs312723761](https://www.ncbi.nlm.nih.gov/projects/SNP/snp_ref.cgi?rs=312723761) | - |
| G4873C | Exon 4 | [rs739888504](https://www.ncbi.nlm.nih.gov/projects/SNP/snp_ref.cgi?rs=739888504) | Same sense mutation |
| G5018A | Intron 4 | [rs14489891](https://www.ncbi.nlm.nih.gov/projects/SNP/snp_ref.cgi?rs=14489891) | - |
| C5067T | Intron 4 | [rs14489892](https://www.ncbi.nlm.nih.gov/projects/SNP/snp_ref.cgi?rs=14489892) | - |
| A5330G | Intron 4 | [rs14489896](https://www.ncbi.nlm.nih.gov/projects/SNP/snp_ref.cgi?rs=14489896) | - |
| C5358T | Intron 4 | [rs14489897](https://www.ncbi.nlm.nih.gov/projects/SNP/snp_ref.cgi?rs=14489897) | - |
| T5381A | Intron 4 | [rs14489898](https://www.ncbi.nlm.nih.gov/projects/SNP/snp_ref.cgi?rs=14489898) | - |
| T5400G | Intron 4 | [rs316271214](https://www.ncbi.nlm.nih.gov/projects/SNP/snp_ref.cgi?rs=316271214) | - |
| A5409G | Intron 4 | [rs316578408](https://www.ncbi.nlm.nih.gov/projects/SNP/snp_ref.cgi?rs=316578408) | - |
| A5523G | Intron 4 | [rs313682508](https://www.ncbi.nlm.nih.gov/projects/SNP/snp_ref.cgi?rs=313682508) | - |
| A5542G | Intron 4 | rs31654137 | - |
| C5571T | Intron 4 | [rs316170953](https://www.ncbi.nlm.nih.gov/projects/SNP/snp_ref.cgi?rs=316170953) | - |
| C5608T | Exon 5 | [rs735820819](https://www.ncbi.nlm.nih.gov/projects/SNP/snp_ref.cgi?rs=735820819) | Same sense mutation |
| C5818T | Exon 5 | [rs317682933](https://www.ncbi.nlm.nih.gov/projects/SNP/snp_ref.cgi?rs=317682933) | F - L |
| G6058A | Exon 5 | [rs741034658](https://www.ncbi.nlm.nih.gov/projects/SNP/snp_ref.cgi?rs=741034658) | R - S |
| C6067T | Exon 5 | - | Same sense mutation |
| A6163G | 3' UTR | [rs316638241](https://www.ncbi.nlm.nih.gov/projects/SNP/snp_ref.cgi?rs=316638241) | - |
| G6768A | 3' UTR | [rs733803434](https://www.ncbi.nlm.nih.gov/projects/SNP/snp_ref.cgi?rs=733803434) | - |
| C6921G | 3' Flanking Region | [rs31472622](https://www.ncbi.nlm.nih.gov/projects/SNP/snp_ref.cgi?rs=314726221) | - |
| G6938A | 3' Flanking Region | [rs732956621](https://www.ncbi.nlm.nih.gov/projects/SNP/snp_ref.cgi?rs=732956621) | - |
| C6988T | 3' Flanking Region | [rs735311160](https://www.ncbi.nlm.nih.gov/projects/SNP/snp_ref.cgi?rs=735311160) | - |
| A7137G | 3' Flanking Region | [rs732483861](https://www.ncbi.nlm.nih.gov/projects/SNP/snp_ref.cgi?rs=732483861) | - |
