# Supplementary material for: Feed conversion ratio, residual feed intake and cholecystokinin type A receptor gene polymorphisms are associated with feed intake and average daily gain in a Chinese local chicken population
Source: J Anim Sci Biotechnol. 2018 Jun 14;9:50. doi: 10.1186/s40104-018-0261-1 (PMC6000933; doi:10.1186/s40104-018-0261-1)
Supplement: Supplementary file 3 — Table S3. SNP genotypes, allelic frequencies and HWE. (DOCX 18 kb) [file 40104_2018_261_MOESM3_ESM.docx]

**Table S3.** SNP genotypes, allelic frequencies and HWE

| **SNP** | **Genotype** | | | **Allele frequencies** | ***P* –values^1^** |
| --- | --- | --- | --- | --- | --- |
| G176A | AA (n = 204) | AG (n = 239) | GG (n = 84) | A = 0.614  G = 0.386 | 0.319 |
| G219A | AA (n = 147 | AG (n = 268) | GG (n = 112) | A = 0.533  G = 0.467 | 0.246 |
| C334A | AA (n = 59) | AC (n = 220) | CC (n = 248) | A = 0.321  C = 0.679 | 0.923 |
| C448T | TT (n = 38) | CT (n = 221) | CC (n = 268) | T = 0.292  C = 0.708 | 0.685 |
| G1290A | AA (n = 145) | AG (n = 263) | GG (n = 119) | A = 0.525  G = 0.475 | 0.990 |
| T3325C | CC (n = 176) | CT (n = 248) | TT (n = 103) | C = 0.569  T = 0.431 | 0.861 |
| C5818T | TT (n = 50) | CT (n = 232) | CC (n = 245) | T = 0.315  C = 0.685 | 0.644 |
| G6058A | AA (n = 34) | AG (n = 192) | GG (n = 301) | A = 0.247  G = 0.753 | 0.651 |
| A6163G | GG (n = 50) | AG (n = 197) | AA (n = 280) | G = 0.282  A = 0.718 | 0.079 |
| G6768A | AA (n = 5) | AG (n = 113) | GG (n = 409) | A = 0.117  G = 0.883 | 0.358 |

^1^, Chi-square test *P*-values.
